# Transferable Ion Force Fields in Water from a Simultaneous Optimization of Ion Solvation and Ion–Ion Interaction

**DOI:** 10.1021/acs.jpcb.1c05303

**Published:** 2021-07-22

**Authors:** Philip Loche, Patrick Steinbrunner, Sean Friedowitz, Roland R. Netz, Douwe Jan Bonthuis

**Affiliations:** † Fachbereich Physik, Freie Universität Berlin, 14195 Berlin, Germany; ‡ Department of Materials Science and Engineering, Stanford University, Stanford 94305, California, United States; § Institute of Theoretical and Computational Physics, Graz University of Technology, 8010 Graz, Austria

## Abstract

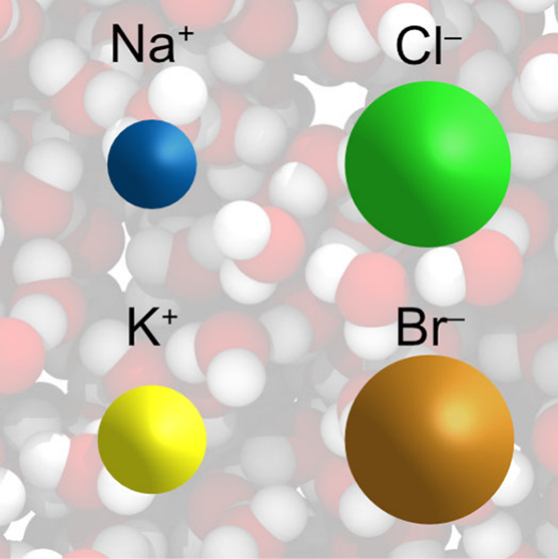

The poor performance of many existing nonpolarizable ion force fields is typically blamed on either the lack of explicit polarizability, the absence of charge transfer, or the use of unreduced Coulomb interactions. However, this analysis disregards the large and mostly unexplored parameter range offered by the Lennard-Jones potential. We use a global optimization procedure to develop water-model-transferable force fields for the ions K^+^, Na^+^, Cl^–^, and Br^–^ in the complete parameter space of all Lennard-Jones interactions using standard mixing rules. No extra-thermodynamic assumption is necessary for the simultaneous optimization of the four ion pairs. After an optimization with respect to the experimental solvation free energy and activity, the force fields reproduce the concentration-dependent density, ionic conductivity, and dielectric constant with high accuracy. The force field is fully transferable between simple point charge/extended and transferable intermolecular potential water models. Our results show that a thermodynamically consistent force field for these ions needs only Lennard-Jones and standard Coulomb interactions.

## Introduction

Aqueous electrolyte solutions not only play an important role for all living organisms but also have wide electrochemical applications with many economical and environmental advantages compared to nonaqueous solvents.^1^ One prominent method to investigate the properties of aqueous solutions is the use of force-field molecular dynamics simulations. The accuracy of these simulations clearly relies on the force field—the interatomic potential used to model the interactions between cations, anions, and water molecules. Besides the Coulomb interaction between charges, the simplest force fields consist of a Lennard-Jones potential to reproduce the Pauli repulsion between overlapping electron shells as well as the London dispersion. Although the latter term models induced dipole–dipole interactions and thus implicitly accounts for the atomic polarizability and ionization potential, these models are referred to as nonpolarizable due to their lack of explicit polarizability. Commonly used nonpolarizable ion force fields include those by Smith,^2,3^ Dang,^4^ and the Groningen Molecular Simulation (GROMOS) set^5^ for the simple point charge/extended (SPC/E) water model and the Assisted Model Building with Energy Refinement (AMBER),^6^ the Chemistry at Harvard Macromolecular Mechanics (CHARMM),^7^ and optimized versions based on these^8^ for the transferable intermolecular potential (TIP) water model family. Yet these force fields produce conflicting results for a number of important systems, including DNA^9,10^ and lipid membranes,^11^ and must be amended for proteins.^12^

The unreliable results have triggered a fierce and ongoing debate about the treatment of polarizability.^13−15^ In particular, since the dielectric environment changes with the ion concentration, the introduction of an explicit ionic polarizability is often considered necessary. Possible implementations include inducible point dipoles, the use of Drude oscillators,^16^ and, more recently, a method based on scaling the Coulomb interaction.^17,18^ What these solutions have in common is the introduction of one or several parameters in addition to the existing Lennard-Jones parameters. Yet the Lennard-Jones potential already provides a large parameter space: Even using a predetermined water force field with a single Lennard-Jones interaction site, there are 10 independent interaction parameters available for a single type of monatomic salt in water (two parameters each for the cation–water, anion–water, cation–anion, cation–cation, and anion–anion interactions). Claims about the necessity of including an explicit polarizability in force fields have been made without attempting an optimization of nonpolarizable ion force fields in the complete parameter space.^15,19^ In fact, it has been shown that classical nonpolarizable force fields for most monovalent and divalent ions can be optimized to reproduce a number of bulk macroscopic thermodynamic observables^20−22^ and that these force fields accurately reproduce the air–water surface affinity.^23^ Nevertheless, whereas in most force fields the number of free parameters is reduced by inferring the parameters of the heterogeneous atom pairs from those of the homogeneous pairs, these so-called mixing rules must be modified for the thermodynamically optimized force fields. Moreover, the parameters of a number of ions have been selected as a reference for the optimization of the other ions,^20^ equivalent to an extra-thermodynamic assumption.

A further point of dispute is the transferability of the ion force fields between water models. The most common nonpolarizable water models are the SPC/E and members of the TIP family. One of the newest offsprings of this family is the TIP4P/ε, which accurately reproduces the dielectric constant over a wide temperature range.^24^ A previous attempt to construct thermodynamically consistent ion force fields for the TIP3P water model only yielded satisfactory behavior at ion concentrations used in the optimization^25^ [Section S8].

Here, we introduce a classical nonpolarizable force field for K^+^, Na^+^, Cl^–^ and Br^–^ optimized for the SPC/E water model, the parameters of which are directly transferable to other major water models, in particular, TIP3P, TIP4P/ε, and, to a lesser degree of accuracy, TIP4P. The force field is optimized with respect to the solvation free energy of an ion pair and the activity coefficient at finite salt concentrations.^20,22^ In contrast to previous work, we apply only Lorentz–Berthelot mixing rules, and by simultaneously optimizing the parameters of all four ion types, no ion parameters need to be fixed in advance. The resulting force field exhibits excellent agreement with the experimental density, ionic conductivity, and dielectric constant as a function of concentration up to 5 mol kg^–1^. Compared with the force fields by Smith and Dang^2^ used for the SPC/E water model and the CHARMM force field^26^ used for the TIP family we find a significantly better agreement with experimental observables.

## Methods

Our simulation systems can be divided into two classes, namely, (1) infinite dilution systems with a single solvated ion and (2) finite concentration simulations. In the infinite dilution systems, a single ion is placed in a cubic box with a box length of *L* = 2.5 nm containing 509 water molecules. For systems at finite dilution we use a box length of *L* = 6.5 nm with different numbers of ion pairs. Each system is first energy-minimized using the steepest descent algorithm and then equilibrated for 200 ps in the *NPT* ensemble at 1 bar and 300 K. For the systems at an infinite dilution we simulate for at least 1 ns, and for the systems at a finite concentration the simulation goes for at least 20 ns.

All simulations are performed using the 2019 version of the GROMACS simulation package^27^ with a 2 fs time step. The velocity rescale thermostat, including a stochastic factor,^28^ is employed with a time constant of 0.5 ps. For the pressure coupling we apply the Berendsen barostat^29^ with a time constant of 1 ps. A cutoff of 0.9 nm is used for the Lennard-Jones interaction, without a long-range dispersion correction.^30^ The Lennard-Jones potential is shifted by its value at the cutoff. Long-range electrostatic interactions are handled using the smooth particle mesh Ewald method (SPME).^31^ In all simulations we use the Lorentz–Berthelot mixing rules, given by σ_*ij*_ = (σ_*i*_ + σ_*j*_)/2 and .

### Solvation Free Energies

The solvation free energy *F* is obtained in the *NVT* ensemble using a two-stage thermodynamic integration method.^32^ First, all Lennard-Jones interactions between the ion and other atoms are gradually turned on; second, the charge of the ion is increased from *q* = 0 to ±*e*, with *e* being the elementary charge. The integration is performed along the reaction coordinate λ, where λ = 0 corresponds to the initial state (A), and λ = 1 corresponds to the final state (B). For the integration, the Hamiltonian is interpolated linearly, *H* = (1 – λ)*H*_A_ + *λH*_B_. The Lennard-Jones and charging transformations are divided into 10 steps each. Free energy differences are calculated by integrating ⟨∂*H*/∂λ⟩ from λ = 0 to λ = 1 using the alchemical-analysis toolkit.^32^ For the integration of the Lennard-Jones potential, we use a soft-core potential to prevent a singularity at λ = 0,^33^ with a soft-core radius α = 0.5 nm and a soft-core power *p* = 1.^27^ The simulation time for each λ state is 1 ns. The simulated free energy *F*_sim_ must be corrected for the effects of the periodic boundary conditions in combination with the Ewald summation as well as for the effect of compressing an ideal gas,
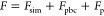
1The first correction reads^34^
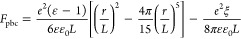
2where ξ is the Wigner constant, given by −2.837 297, ε is the dielectric constant of the water model, *L* is the length of the cubic box, and *r* = 2^1/6^σ is the ion’s Lennard-Jones radius. The first term in eq 2 stems from the interaction of the ion with its periodic images, and the second term is derived from effects of the homogeneous background charge. For our system the correction from eq 2 equals ∼1 *k*_B_*T*. The second correction equals
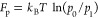
3resulting from the fact that the experimental free energy refers to a transfer of an ideal gas at pressure *p*_0_ = 1 atm into a 1 mol/*l* ideal solution. Using *p*_1_ = *k*_B_*Tn*, with *n* being the number density, we find *F*_p_ = 3.2*k*_B_*T*. The experimental free energies of ion pairs at 300 K were calculated from Marcus^35^ and Tissandier et al.;^36^ see section S2 in the Supporting Information for details.

### Ionic Activity Coefficients

The activity coefficients are obtained using Kirkwood-Buff integrals. With charge neutrality, the monovalent ion number density *n* = *n*_+_ = *n*_–_ can be expressed in terms of Kirkwood-Buff integrals *G*_*αβ*_^∞^ as *n* = (*G*_+–_^∞^ – *G*_++_^∞^)^−1^.^37^ Therefore, the logarithmic derivative of the mean activity *a* with respect to *n* equals the following combination of Kirkwood Buff integrals
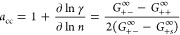
4where *+*, −, and *s* denote cation, anion, and solvent, respectively, and γ = *a*/*n* denotes the mean molar activity coefficient of anions and cations. The Kirkwood-Buff integrals are calculated from pair correlation functions *g*_αβ_ (*r*_1_, *r*_2_) according to^38^
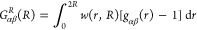
5using a geometrical weight function
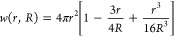
6after which *G*_*αβ*_^∞^ is obtained by a linear extrapolation of *G*_*αβ*_^*R*^(*R*) as a function of 1/*R* to 1/*R* = 0. Experimental activities are obtained from Hamer and Wu.^39^

### Ionic Conductivity

According to the Einstein–Smoluchowski relation the conductivity κ of monovalent ions is given by
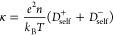
7where *D*_self_^+^ and *D*_self_^–^ denote the cationic and anionic self-diffusivities, respectively. For the self-diffusion coefficient *D*_self_ we use the same simulation trajectories as for the activity coefficients. The coefficients are obtained from a linear fit to the long-time mean-squared displacement (MSD) (see section S5 in the Supporting Information)
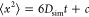
8where the constant *c* accounts for short-time deviations. To account for finite size effects, we use the relation
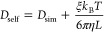
9where *D*_sim_ is the simulated self-diffusion coefficient, and η is the viscosity of the water model,^40^ taken from González and Abascal.^41^

### Dielectric Constant at Finite Salt Concentration

We obtain the dielectric decrement by employing a linear response formalism for salt solutions.^42^ In this approach, the total electric susceptibility spectrum χ(ω) is decomposed into three additive contributions
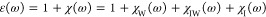
10that are related to time correlation functions between the water dipole moment, water dipole-ion current, and ion-current, respectively. The dielectric constant is then obtained from taking the limit ω → 0; see section S6.

## Results and Discussion

To find the optimal parameters we start by choosing Lennard-Jones parameters for the chloride ion. We then pick a set of cation parameters that lie on the solvation free energy isolines so that they reproduce the experimental solvation free energy of KCl and NaCl. For each partial parameter set (Na^+^, K^+^, Cl^–^), we then calculate the log–log derivative *a*_cc_ of the activity for several concentrations and calculate the mean-squared deviation of *a*_cc_ from the experimental activity derivatives. For the best parameter set of chloride and the cations, we repeat the optimization for the bromide salts (KBr, NaBr) while varying the Br^–^ parameters and keeping the cation parameters fixed. With this strategy, the optimal parameter set only depends on the initial parameters of the chloride ion. We repeat the procedure for different choices of the Cl^–^ parameters. See sections S1 and S3 in the Supporting Information for additional details.

**Figure 1:**
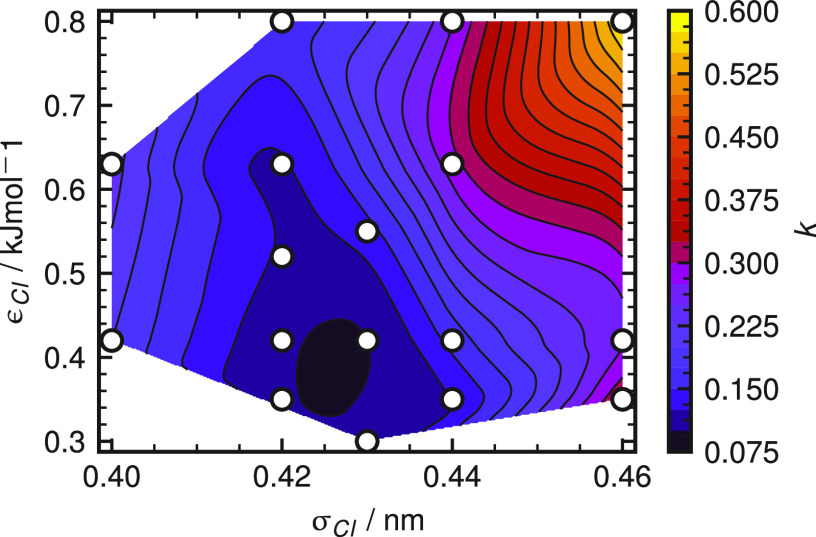
Mean squared difference *k* defined in eq 11 between the simulated *a*_cc_ and experimental^39^ activity derivatives *a*_cc,exp_ for the optimal parameters of the four salts, as a function of the Lennard-Jones parameters of the Cl^–^ ion. Circles depict the parameter combinations for which the simulations were performed, and the contour map is calculated by a cubic interpolation.

Figure 1 shows the mean-squared deviation
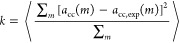
11from the experimental activity derivatives *a*_cc,exp_ for all our tested initial chloride parameters. In eq 11 the ⟨ · ⟩ term denotes the mean over all salts, and *m* indicates the molality, which is considered in the range of 0 < *m*, mol/kg < 5. We find a minimum in this σ–ϵ landscape for σ_Cl_ = 0.43 nm and ϵ_Cl_ = 0.42 kJ mol^–1^. Our cubic interpolation suggests the minimum to be at slightly smaller σ and ϵ values. However, the effect of these small changes in the parameters on the activity coefficients can not be resolved with sufficient accuracy. Using these Lennard-Jones parameters we obtain our optimal ion parameters as shown in Table 1. Note that the third digit of the σ_*i*_ parameter of sodium is important; even tiny changes in the sodium parameters have drastic effects on the activities (see Figure S3 in the Supporting Information).

**Table 1:** Optimal Ion Lennard-Jones Parameters and Charges^a^

ion	σ_*i*_ (nm)	ϵ_*i*_ (kJ mol^–1^)	q (e)
K^+^	0.283	0.90	+1
Na^+^	0.231	0.45	+1
Cl^–^	0.43	0.42	–1
Br^–^	0.443	0.75	–1

a Lorentz-Berthelot mixing rules are used for σ_*ij*_ and ϵ_*ij*_. The Lennard-Jones parameters of the used water models are listed in table S4 in the Supporting Information.

**Figure 2:**
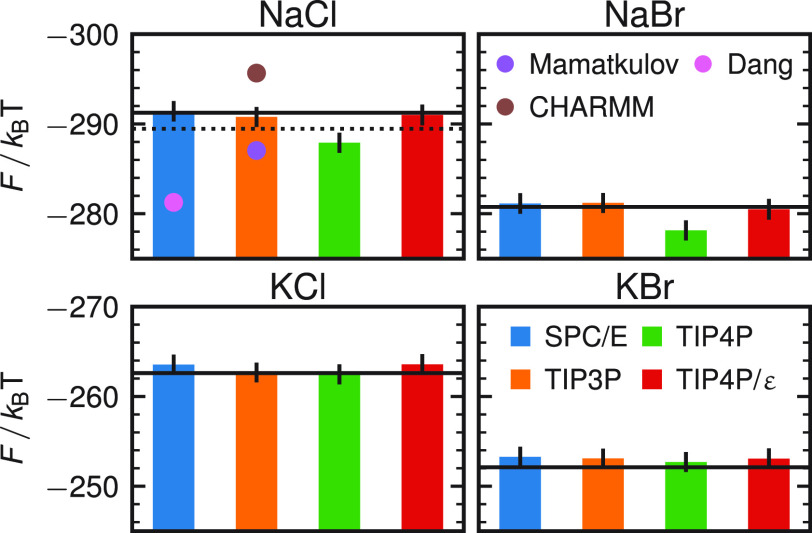
Salt solvation free energies for ion parameters using different water models. Experimental free energies at 300 K (solid black lines) are calculated from Marcus^35^ and Tissandier et al.^36^ Symbols show the solvation free energy of reported force fields.^2,25,26^ The dotted black line corresponds to the solvation free energy of NaCl used in our previous work as well as in the work of Mamatkulov and Schwierz;^25^ see section S2 of the Supporting Information.

Next, we test the transferability of our parameters to water models other than SPC/E. Figure 2 shows the solvation free energy for the four salts and the four water models. All free energies agree within 2% with the experimental values taken from refs (35) and (36). We find that changing the water model has a negligible effect on the solvation free energies. Symbols show the solvation free energy of the force fields from refs (2, 25, and 26).

**Figure 3:**
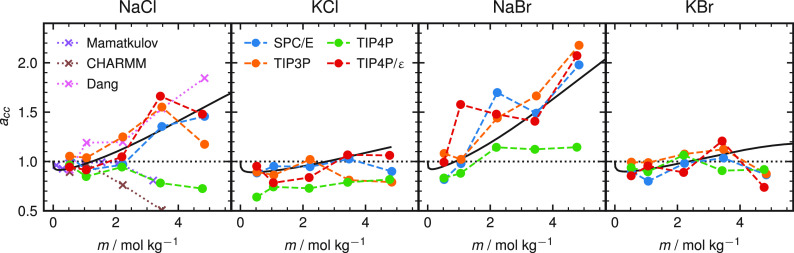
Activity derivative according to eq 4 of NaCl, KCl, NaBr, KBr as a function of the salt concentration. Different colors depict different water models. The cross symbols denote results from the force fields by Smith and Dang^2^ (SPC/E, pink), CHARMM^26^ (TIP3P, brown), and reproduced from Mamatkulov and Schwierz^25^ (TIP3P, purple). Solid black lines depict the experimental activity derivatives.^39^ Errors are between 0.1 and 0.3 (estimated using a block averaging with five blocks; see Figure S4 for individual error bars).

Figure 3 shows the activity derivative *a*_cc_ for the four different water models by applying eq 4. For a selection of concentrations, the radial distribution functions are shown in section S4 in the Supporting Information. We also show activities for NaCl using parameters from two common force fields, by Smith and Dang^2^ (pink crosses) for the SPC/E water model and CHARMM^26^ (brown crosses) optimized for the TIP family, as well as the newer force field by Mamatkulov and Schwierz^25^ optimized for TIP3P (purple crosses). As shown in Figure 3, we find good agreement between our force field results and experiments for all water models, except for the sodium salts in the TIP4P water model. This shows that the same ion force fields can be used in combination with all major nonpolarizable water models, in contrast to previous suggestions.^25^ In general, we find that the agreement between all water models is better for the potassium salts compared to the sodium salts. Our optimization also shows that the potassium salts are more robust with regard to a variation of the parameters (Figure S3 of the Supporting Information).

**Figure 4:**
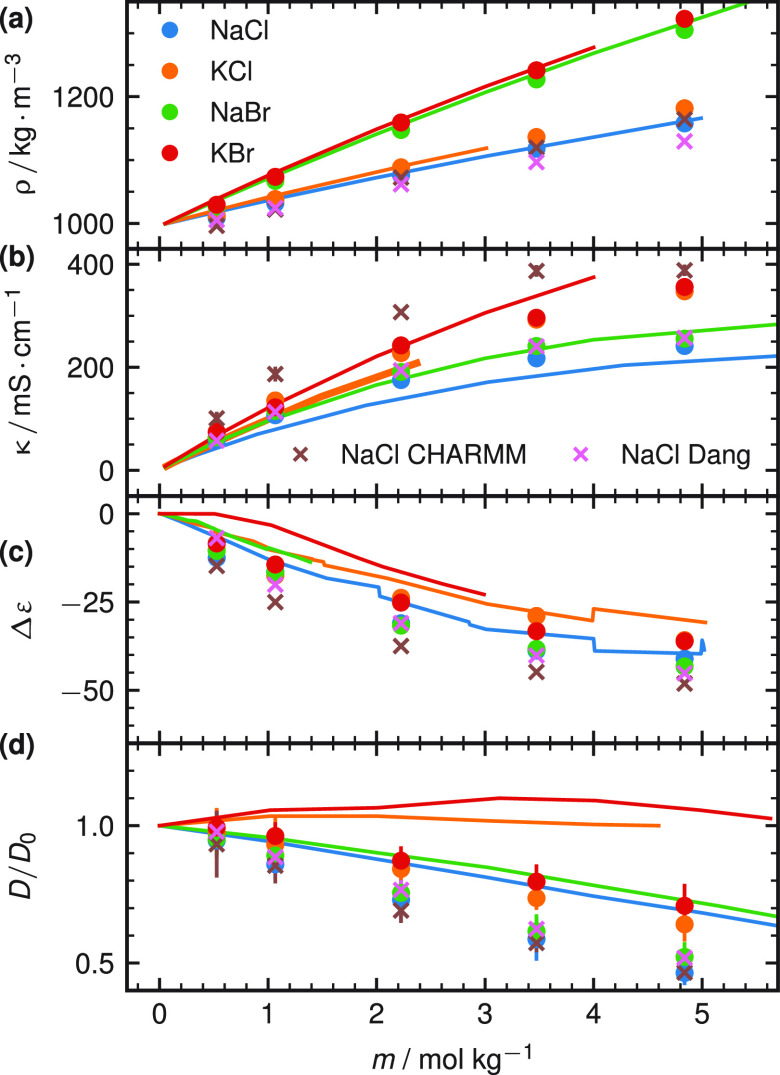
(a) Simulated mass density ρ for the four salts using the optimized force field (●) and literature force fields^2,26^ (×) together with the experimental densities^43,44^ (solid lines) as a function of the salt concentration. (b) Ionic conductivities κ together with the experimental values (solid lines).^43,45^ (c) Dielectric decrement Δε together with the experimental values (solid lines).^46−53^ (d) Water diffusion constant normalized by its value for pure water together with the experimental values (solid lines).^54^ The SPC/E water model is used in all panels. Results from other water models are shown in Figure S8.

Finally, we test the ion parameters against a number of experimental observables that we did not optimize for. In Figure 4, we show the mass density ρ, the conductivity κ (eqs 7 and 8), the dielectric decrement Δε (eq 10), and the water self-diffusion constant *D* (eqs 8 and 9). The density increment in Figure 4a shows excellent agreement with the experimental data. The higher density of the bromide salts is due to the much higher molar mass of bromide (*m*_Br_ = 79.90 u) compared to chloride (*m*_Cl_ = 35.45 u). The conductivity in Figure 4b shows a quantitative agreement with the experimental data for all salts up to at least 2 mol/kg. A selection of MSDs are shown in section S5 in the Supporting Information. We find that the sodium salts have a lower conductivity when compared to the potassium salts. Because of the small size of sodium, it exhibits a strong hydrogen bonding to water molecules, reducing its diffusion constant compared to potassium. This effect is faithfully reproduced by our new force field. The dielectric decrement Δε, displayed in Figure 4c, is obtained by subtracting the bulk water dielectric constant for SPC/E, ε_SPC/E_ = 72.0, from the dielectric constant calculated using eq 10. The experimental values are taken from refs (46−53) and have been averaged for each salt type. Both the trend and the amplitude of Δε are accurately captured by our new force field. The water diffusion coefficient, calculated from eqs 8 and 9, is shown in Figure 4d. Again, the order of the ions is well-reproduced. However, the simulated water diffusivity for the potassium salts fails to capture the experimental trend as a function of the salt concentration. This issue has been noticed for rigid nonpolarizable water models before.^55^ To compare with other NaCl force fields, we show the results for Smith and Dang^2^ and CHARMM^26^ as pink and brown symbols in Figure 4a–d, respectively. Overall, our new force field agrees better with the experimental NaCl data, but note that the density using the CHARMM^26^ parameters coincides with our results. For the other water models, the observables show a very similar behavior (see Figure S8 of the Supporting Information), confirming the transferability of the ion parameters between the water models.

## Conclusion

We have optimized a nonpolarizable force field for aqueous solutions of NaCl, KCl, NaBr, and KBr up to concentrations of 5 mol/kg. In contrast to previous work, all Lennard-Jones parameters are determined rigorously by simultaneously optimizing four ion pairs with respect to the experimental solvation free energy and the activity, in combination with standard mixing rules. This procedure eliminates the necessity to select a reference ion, which turns out to be crucial for the performance of the resulting force field. The force field is fully transferable between the rigid water models SPC/E, TIP3P, and TIP4P/ε. In TIP4P, the activity of the sodium salts is poorly reproduced, which is likely to be related to the inferior dielectric properties of TIP4P.^24^ The previously used modified mixing rules for heterogeneous atom pairs,^20^ although perfectly compatible with the current optimization strategy, are unnecessary for these ions. Our new force field reproduces the dependence of the density, the conductivity, and the dielectric decrement on the salt concentration over the entire concentration range, but not the water self-diffusion constant. The successful optimization shows that an explicit polarizability is unnecessary despite the strong variation of the dielectric constant with the salt concentration. Instead, standard Coulomb and Lennard-Jones interactions are sufficient to accurately capture the macroscopic thermodynamics of aqueous ionic solutions, as well as the conductivity and the dielectric constant. Note that more complex water and ion models might be necessary to capture other kinetic properties, such as the water self-diffusion coefficient.^55^ In conclusion, the newly optimized force field ensures that the electrolyte thermodynamics are accurately reproduced in simulations with the most widely used water models without introducing a more complex interaction potential.

## Supporting Information Available

Details of the optimization procedure, calculation of the diffusivity and the dielectric decrement, observables for different water models, and discussion of the comparison to other force fields (PDF)

## Notes

Data Availability Original simulation and analysis files are available at http://dx.doi.org/10.17169/refubium-30888.
