# Supplementary material for: Transferable Ion Force Fields in Water from a Simultaneous Optimization of Ion Solvation and Ion–Ion Interaction
Source: J Phys Chem B. 2021 Jul 22;125(30):8581–7. doi: 10.1021/acs.jpcb.1c05303 (PMC8389903; doi:10.1021/acs.jpcb.1c05303)
Supplement: Supplementary file 1 — jp1c05303_si_001.pdf [file jp1c05303_si_001.pdf]

Transferable Ion Force Fields in Water From Simultaneous Optimization of Ion Solvation and Ion-Ion Interaction: Supplementary Information

Philip Loche,† Patrick Steinbrunner,† Sean Friedowitz,‡ Roland R. Netz,† and Douwe Jan Bonthuis¶

†*Fachbereich Physik, Freie Universität Berlin, 14195 Berlin, Germany*

‡*Department of Materials Science and Engineering, Stanford University, Stanford, California 94305, United States*

¶*Institute of Theoretical and Computational Physics, Graz University of Technology, 8010 Graz, Austria*

Contents

S1 Optimization strategy **S2**

S2 Experimental solvation free energy **S3**

S3 Free energy isolines **S6**

S4 Radial Distribution functions **S8**

S5 Fitting the MSD **S11**

S6 Details on calculating the dielectric decrement **S11**

S7 Observables for different water models **S12**

S8 Comparison to other force fields **S12**

References **S14**

S1 Optimization strategy

The goal of the optimization strategy is to find the chloride parameters in a way that reproduces the experimental solvation free energy of every cation/anion pair, and provides the best fit to the logarithmic activity derivative a_{cc} as a function of concentration. Therefore, we start by selecting one set of parameters for the chloride ion and calculating the free energy isolines of sodium and potassium. This gives one isoline for sodium and one isoline for potassium for every choice of chloride. The details of the isoline calculation are discussed in Section S3. For a number of points along these isolines, we calculate the logarithmic activity derivative of NaCl and KCl as a function of concentration. To avoid having to calculate a_{cc} curves of NaBr and KBr for every possible choice of sodium and potassium parameters, we first perform a partial optimization of the sodium and potassium parameters for every choice of chloride parameters. To that end, we calculate the partial square deviation k' (defined identically to Eq. (11) from the main text) for each of the a_{cc} curves, by summing the square deviation over all concentrations used, but only over the salts NaCl and KCl. This gives us one set of best-performing sodium and potassium parameters for every initial choice of chloride parameters. For these best-performing sodium and potassium parameters, we calculate the logarithmic activity derivative of NaBr and KBr as a function of concentration for a number of points along the NaBr and KBr free energy isolines. We now optimize the bromide parameters by calculating the square deviation k (Eq. (11) from the main text), summing over the concentrations used and over all four salt combinations. This gives us one value of k for every choice of chloride parameters, corresponding to the total square deviation from the experimental activity derivatives when using the best possible sets of sodium, potassium, and bromide parameters. We find the final optimized force field by repeating the scheme above for different choices of the initial chloride parameters.

S2 Experimental solvation free energy

The solvation free energy of ion pairs is measured experimentally at a temperature of 298.15 K. A summary of experimental values of the enthalpy ΔH, entropy ΔS and Gibbs free energy ΔG is given in Table S1, in chronological order.

Table S1: Experimental thermodynamic data for the transfer of ion pairs from the 1 atm gas phase to the hypothetical ideal 1 mol/l liquid state at 298.15 K. The reported enthalpy ΔH, entropy ΔS, and free energy ΔG of solvation are converted to kJ/mol (ΔH, ΔG) and J/(mol K) (ΔS). The pressure correction F_p applies to reported transitions from the 1 atm gas phase to a single ion in solution, or from the 1 mol/l gas phase to the ideal 1 mol/l solution. The solvation free energies of the ion pairs are calculated in two ways when possible, and the final column (Dev.) contains the absolute difference between the free energies when calculated from ΔG or when calculated from ΔH and ΔS.

**Ion**	ΔH	ΔS	ΔG	F_p	**Salt**	ΔG	$\Delta H - T\Delta S$	**Dev.**
Latimer[1]								
Cl	-372.4	-71.1	-355.6	7.9	NaCl	-722.6	-721.0	1.6
Br	-338.9	-56.1	-326.4	7.9	KCl	-649.8	-647.9	1.9
Na	-403.8	-60.7	-382.8	7.9	NaBr	-693.3	-692.0	1.3
K	-320.1	-25.1	-310.0	7.9	KBr	-620.5	-618.9	1.6
Noyes[2]								
Cl		-164.0	125.5		NaCl	-719.0		
Br		-147.3	154.4		KCl	-644.6		
Na		-20.4	-844.5		NaBr	-690.1		
K		13.3	-770.1		KBr	-615.7		
Rosseinsky[3]								
Cl	-1469.0	-207.1	-1407.1		NaCl	-728.0	-728.3	0.3
Br	-1454.8	-191.6	-1393.3		KCl	-654.4	-654.4	0.0
Na	685.3	21.3	679.1		NaBr	-714.2	-718.7	4.5
K	769.9	56.9	752.7		KBr	-640.6	-644.8	4.2
Salomon[4]								
Cl	-1465.7		-1402.9		NaCl	-722.6		
Br	-1447.2		-1377.4		KCl	-649.4		
Na	686.6		680.3		NaBr	-696.6		
K	770.3		753.5		KBr	-623.4		
Friedman[5]								
Cl	-340.2	-76.1	-317.1		NaCl	-728.4	-728.7	0.3
Br	-325.9	-60.7	-303.3		KCl	-655.2	-655.6	0.4
Na	-443.9	-109.6	-411.3		NaBr	-714.6	-719.1	4.5
K	-360.2	-74.1	-338.1		KBr	-641.4	-646.0	4.6

Table S1: Continued

Ion	ΔH	ΔS	ΔG	F_p	Salt	ΔG	$\Delta H - T\Delta S$	Dev.
Gomer[6]								
Cl			-340.6		NaCl	-719.8		
Br			-318.4		KCl	-646.5		
Na			-379.2		NaBr	-697.6		
K			-305.9		KBr	-624.3		
Wagman[7]		[1]						
Cl	66.0	-108.7			NaCl		-722.9	
Br	97.5	-92.6			KCl		-651.0	
Na	-849.5	-94.7			NaBr		-696.1	
K	-766.6	-57.8			KBr		-624.3	
Marcus[8]								
Cl		-96.9						
Br		-81.2						
Na		-89.0						
K		-52.1						
Conway[9]								
Cl	-363.6	-79.9	-399.7		NaCl	-727.3	-726.4	0.9
Br	-349.4	-64.4	-325.9		KCl	-653.7	-652.6	1.1
Na	-419.3	-109.7	-387.6		NaBr	-713.5	-716.8	3.3
K	-334.9	-74.2	-314.0		KBr	-639.9	-643.0	3.1
Marcus[10]	[2]	[3]						
Cl	-367	-75	-347		NaCl	-722	-727.5	5.5
Br	-336	-59	-321		KCl	-651	-656.6	5.6
Na	-416	-111	-375		NaBr	-696	-701.3	5.3
K	-334	-74	-304		KBr	-625	-630.3	5.3
Tissandier[11]								
Cl	-1470.0		-1408.7		NaCl	-728.0		
Br	-1439.0		-1381.9		KCl	-656.2		
Na	686.6		680.7		NaBr	-701.2		
K	769.9		752.5		KBr	-629.4		
Fawcett[12]								
Cl	-1469.7	-205.8			NaCl		-727.7	
Br	-1438.8	-190.1			KCl		-655.8	
Na	686.7	20.0			NaBr		-701.4	
K	769.6	56.9			KBr		-629.5	

[1] Calculated from the transfer of the neutral species in gas to the charged specied in solution.
[2] Data from Wagman et al.[7], shifted by $\Delta H = 433.2\,\mathrm{kJ\,mol^{-1}}$ based on the proton hydration enthalpy.
[3] Data from Marcus and Loewenschuss[8], shifted by the proton entropy $\Delta S = -22.2\,\mathrm{J\,K^{-1}mol^{-1}}$.

Table S1: Continued

Ion	ΔH	ΔS	ΔG	F_p	Salt	ΔG	$\Delta H - T\Delta S$	Dev.
Schmid[13]								
Cl	-392	-64.5		7.9	NaCl		-727.6	
Br	-361	-48.7		7.9	KCl		-655.3	
Na	-391	-68.3		7.9	NaBr		-701.3	
K	-308	-32.2		7.9	KBr		-629.0	
Kelly[14]								
Cl			-311.7	7.9	NaCl	-727.6		
Br			-285.8	7.9	KCl	-655.7		
Na			-431.8	7.9	NaBr	-701.7		
K			-359.8	7.9	KBr	-629.7		

Some data sets listed in Table S1 report two independent values and calculate the third, making them thermodynamically consistent automatically, whereas some report three independent values. These data sets are typically not exactly thermodynamically consistent, but the differences are rarely more than 1 k_BT. Exceptions include early estimates of the Br free energy and, notably, the book by Marcus[10]. One possible reason for the inconsistency in Marcus' data is that at least one of his papers reports the 'solvation energy proper', referring to the transfer from the 1 atm gas phase to a single ion in solution, using the wrong sign for the pressure correction[15]. The affected data set also appears to be thermodynamically inconsistent, even though according to the text ΔG is calculated from ΔH and ΔS. This inconsistency might well be due to the sign error, because the deviations are of the order of the value of the pressure correction.

In his book, however, Marcus uses standard states, so the pressure correction is not to blame[10]. It is clear that the enthalpy data is taken from the NBS tables compiled by Wagman et al.[7], shifted by $z(433.2)$ kJ/mol, where z is the ion valency. Taking the entropy change for the transition of the uncharged species in gas to the charged species in solution also from the NBS tables yields values for ΔG that are very close to the listed ΔG values[10]. Yet in 1984, Marcus recalculated the entropy from the Sackur-Tetrode equation, including an electronic contribution based on the population of the different energy levels[8]. These values, shifted by $z(-22.2)$ J/(mol K), are listed in Marcus' book as ΔS.

Interestingly, using ΔH and ΔS from Marcus[10], which corresponds to the transition of the charged species in the gas phase to the charged species in the hypothetical ideal 1 mol/l liquid phase, gives almost exactly the same values as the ones found independently by Tissandier et al.[11]. A subsequent estimate by Fawcett[12] using the same approach for the electronic contribution as Marcus and Loewenschuss[8], as well as estimates by Schmid et al.[13] and Kelly et al.[14] give very similar results as well. Therefore, we believe that the Tissandier/Marcus (ΔH, ΔS) data is probably the most accurate data set available.

Since our simulations – like most molecular dynamics simulations – are performed at a temperature of 300 K, we calculate the solvation free energy ΔG at 300 K from the entropy change ΔS and enthalpy change ΔH. Because the volume change during solvation of an ion

pair is negligible[16], we can compare the simulated solvation free energy F directly to the experimental solvation free energy $\Delta H - T\Delta S$. Averaging the values from Marcus[10] and Tissandier et al.[11] gives the final values listed in Table S2.

Table S2: Reference experimental values for the solvation free energy at T = 300 K in units k_BT. The uncertainty equals the difference between the data from Marcus[10] and Tissandier et al.[11].

**Salt**	$\Delta H - T\Delta S$ (k_BT)	$\pm$
NaCl	-291.6	0.18
KCl	-263.0	0.15
NaBr	-281.0	0.05
KBr	-252.4	0.38

Note that we used the values of ΔG from Marcus[10] at 298.15 K in our previous optimizations[16–18]. These values were also used by Mamatkulov and Schwierz[19]. Given the relatively small deviations, however, these different reference values are generally not expected to have had a major impact on the final force field parameters in those papers.

S3 Free energy isolines

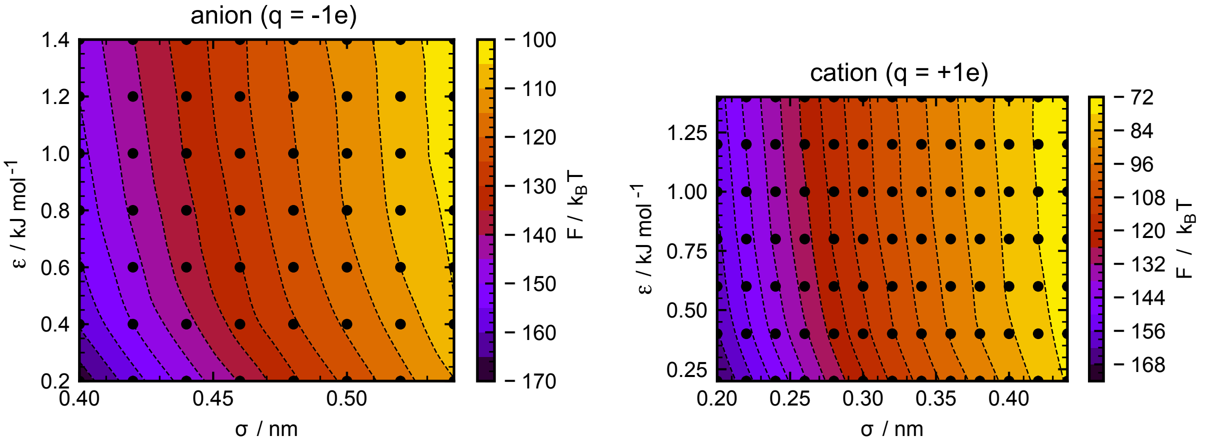

Fig. S1: Solvation free energy (F) of monovalent cations and anions for different σ and ϵ values in SPC/E water. Black dots show the grid on which the simulations where performed. Values between the black dots are obtained using cubic interpolation.

The calculation of the derivatives $\langle \partial H/\partial\lambda \rangle$ of single ions is described in the main text. The single-ion free energy is obtained using Simpson's integration rule. The numerical integration is verified and found to be accurate by comparing to the energy obtained using a 12-point open Gaussian integration scheme. From a thermodynamic point of view, the only relevant quantity is the solvation free energy of ion pairs. The free energy isoline is defined

as the line in parameter space where the simulated solvation free energy of the pair reproduces the experimental value. To obtain the free energy isolines, we calculate the free energy for several single anions and cations. Note that when solvating ion pairs from the vacuum into the bulk water, the electrostatic energy originating in bringing a single ion across the water interface vanishes, and therefore we do not take it into account. Figure S1 shows the resulting solvation free energy for the SPC/E water model. Black dots show the grid on which the simulations where performed and the values between the black dots are obtained using cubic interpolation. Since water is charge-asymmetric it favors anionic versus cationic solvation[20–23]. It is also clear from Fig. S1 that solvation of ions with a smaller σ are favored as predicted by the Born model[24].

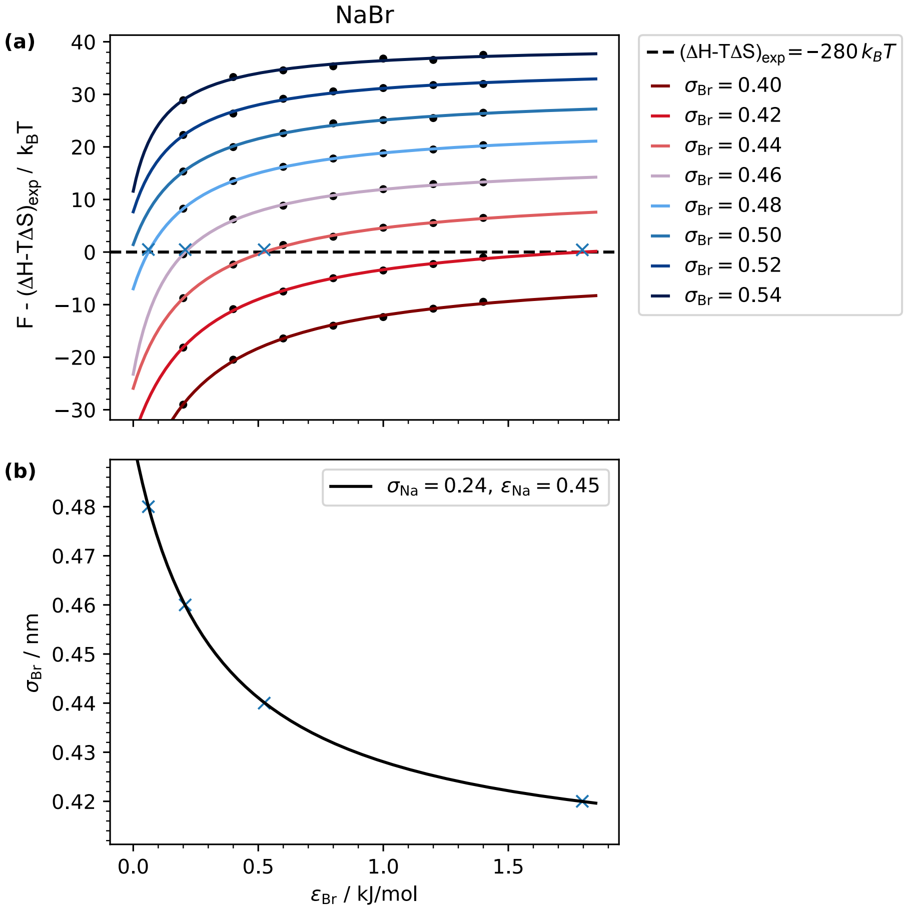

Fig. S2: (a) Solvation free energy F of NaBr in SPC/E water as a function of $\varepsilon_{\mathrm{Br}}$ for fixed $\sigma_{\mathrm{Na}} = 0.239\,\mathrm{nm}$ and $\epsilon_{\mathrm{Na}} = 0.45\,\mathrm{kJ/mol}$. Solid lines show fits according to equation eq. (S1). Different colors show different values of σ_{Br}. Blue crosses mark roots of the fit used for isolines. (b) NaBr isoline for fixed sodium parameters (same as in a). Blue crosses are same as in a. The black solid line is a fit according to eq. (S2).

Now the solvation free energies of ion pairs are compared to the experiment. We fix the parameters of one ion of the pair, and calculate the ion pair solvation free energy as a function of the parameters of the other ion. As an example, Figure S2a shows the solvation

free energy F of NaBr, reduced by the experimental solvation free energy $(\Delta H - T\Delta S)_{\mathrm{exp}}$. Here the sodium parameters are fixed and the bromide parameters are varied. The solid lines are fits according to

$$F(\epsilon) = a - \frac{b}{c\epsilon + 1} \tag{S1}$$

where a, b and c are fit parameters. Different colors denote different values of σ_{Br}. From the roots of the fits (shown as blue crosses), we obtain the isoline, *i.e.* pairs of σ_{Br} and ϵ_{Br} which reproduce the experimental solvation free energy for fixed values of σ_{Na} and ϵ_{Na}. We now fit the isoline according to

$$\sigma(\epsilon) = \frac{f}{\epsilon + d} + e \tag{S2}$$

where d, e, and f are again fit parameters. The isoline and the fit are shown in Figure S2b and the best fit parameters are shown in table S3. Along these isolines, we calculate the activity coefficients.

Table S3: Optimal fit parameters for the isolines according to eq. (S2) **as a function of the parameters of the ion printed in boldface. The parameters of the other ion are fixed to their optimized values.**

**Salt**	$\boldsymbol{d}$ $\left(\frac{\mathrm{kJ}}{\mathrm{mol}}\right)$	$\boldsymbol{e}$ (nm)	$\boldsymbol{f}$ $\left(\mathrm{nm}\,\frac{\mathrm{kJ}}{\mathrm{mol}}\right)$
**Na**Cl	0.4100	0.1973	0.0286
**K**Cl	0.2380	0.2680	0.0173
Na**Br**	0.2611	0.4209	0.0222
K**Br**	0.2613	0.4225	0.0223

In Figure S3 we show the activity derivatives a_{cc} for several parameter sets using the SPC/E water model. Note that all parameters shown reproduce the experimental solvation free energy as they are all drawn from isolines. However, only a few also agree with the experimental activities. The best parameter sets are shown as solid dots. As mentioned in the main text we find that especially the sodium chloride activity is sensitive to changes in the Lennard Jones parameters.

In Fig. S4, we show the estimates of the error of the individual values of a_{cc} for the optimal force field parameters.

S4 Radial Distribution functions

We show the radial distribution functions of the optimized force field at two different concentrations in Fig. S5.

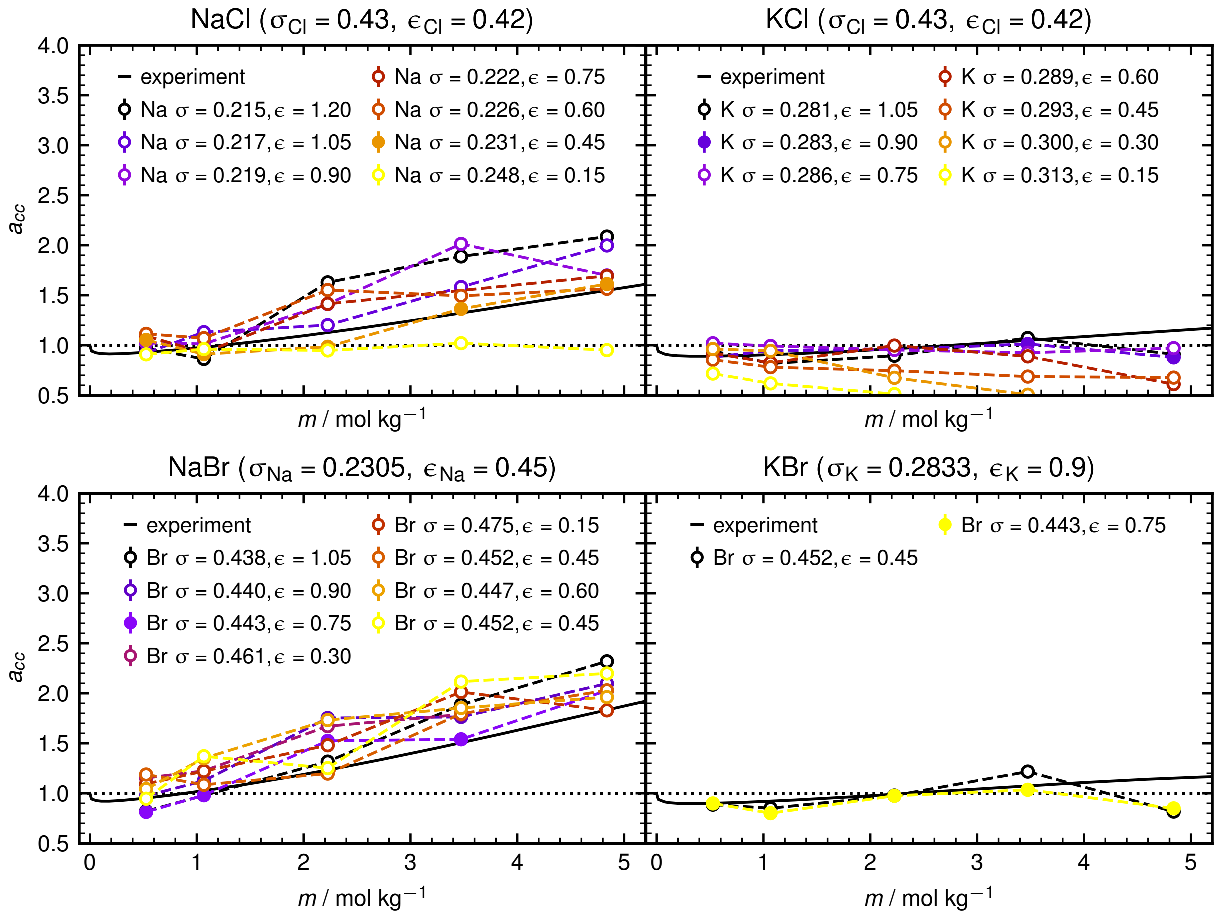

Fig. S3: Activity derivatives according to eq. (4) in the main text of NaCl, KCl, NaBr, KBr as a function of the salt concentration using the SPC/E water model. Different colors depict different ion parameters. Solid symbols show parameter sets with the smallest mean deviation from the experimental activity.

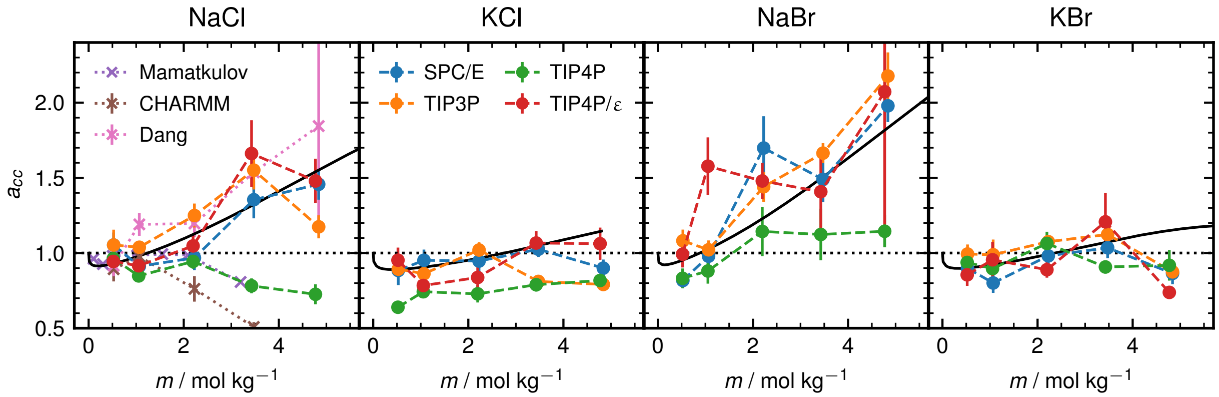

Fig. S4: Activity derivatives of the optimized force field parameters (identical to the data shown in Fig. 3), including error bars of the simulation data. Errors are estimated using a block averaging with 5 block where each block is at least 4 ns.

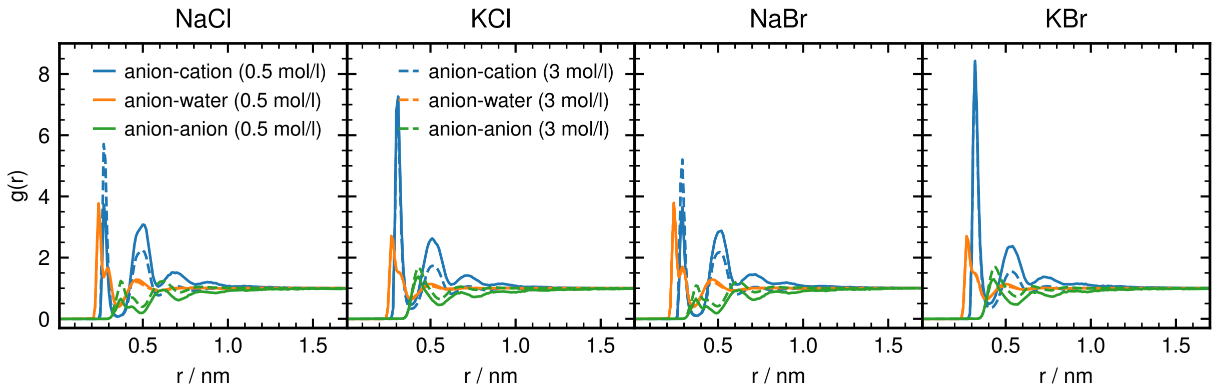

Fig. S5: Anion-cation (blue), anion-water (orange) and anion-anion (green) radial distribution functions $g(r)$ using the SPC/E water model. Solid lines show a concentration of 0.5 mol/l and dashed lines 3 mol/l.

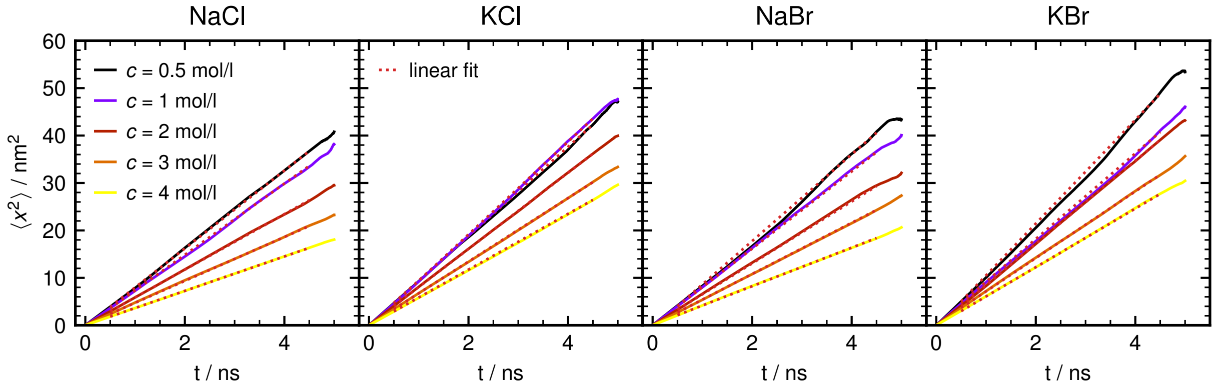

Fig. S6: Mean square displacement $\langle x^2 \rangle$ of the ions using the SPC/E water model, averaged over anions and cations. Different colors refer to different concentrations. Diffusion constants are obtained by a linear fit from 0.5 to 4.5 ns shown as red dotted lines

S5 Fitting the MSD

We show the mean square displacement of the ions, summed over cations and anions, as a function of time in Fig. S6. The diffusion constants $D_{\mathrm{self}}^{+} + D_{\mathrm{self}}^{-}$ are obtained from linear fits shown as red dotted lines in Fig. S6.

S6 Details on calculating the dielectric decrement

We follow the procedure detailed in Ref. 25 to obtain the dielectric spectra from each salt simulation. This procedure begins with the linear response relationship between the electric susceptibility $\chi(\omega)$ and the equilibrium auto-correlation function for fluctuations of the polarization $M(t)$,

$$\chi(\omega) = -\frac{1}{3Vk_{\mathrm{B}}T\epsilon_0}\int_0^\infty e^{-i\omega t}\frac{\mathrm{d}}{\mathrm{d}t}\langle M(0)\cdot M(t)\rangle\,\mathrm{d}t. \tag{S3}$$

For our system the total instantaneous polarization is the sum of two contributions $M(t) = M_{\mathrm{W}}(t) + M_{\mathrm{I}}(t)$ for the water molecules and ions, respectively. However, in a periodic simulation cell, it is convenient to express the ionic polarization contribution in terms of the ionic current $J = \mathrm{d}M_{\mathrm{I}}/\mathrm{d}t$ due to toroidal shifts that occur when charged particles traverse the simulation boundaries[26]. Manipulating the above expression, we can express $\chi(\omega)$ in terms of time correlations between these two contributions to the total polarization, and arrive at

$$\chi(\omega) = \chi_{\mathrm{W}}(\omega) + \chi_{\mathrm{IW}}(\omega) + \chi_{\mathrm{I}}(\omega). \tag{S4}$$

The first term $\chi_{\mathrm{W}}(\omega)$ is due to the water dipole moment correlations and is given by,

$$\chi_{\mathrm{W}}(\omega) = \phi_{\mathrm{W}}(0) - i\omega\int_0^\infty e^{-i\omega t}\phi_{\mathrm{W}}(t)\,\mathrm{d}t, \tag{S5}$$

where we have defined the water dipole time correlation function as

$$\phi_{\mathrm{W}}(t) = \frac{\langle M_{\mathrm{W}}(0)\cdot M_{\mathrm{W}}(t)\rangle}{3Vk_{\mathrm{B}}T\epsilon_0}. \tag{S6}$$

The second term $\chi_{\mathrm{IW}}(\omega)$ arises from cross-correlations between the water dipoles and salt ion currents. This term can ultimately be expressed as

$$\chi_{\mathrm{IW}}(\omega) = -2\int_0^\infty e^{-i\omega t}\phi_{\mathrm{IW}}(t)\,\mathrm{d}t, \tag{S7}$$

with the ion-water cross-correlation function defined as

$$\phi_{\mathrm{IW}}(t) = \frac{1}{2}\frac{\langle M_{\mathrm{W}}(0)\cdot J_{\mathrm{I}}(t) - J_{\mathrm{I}}(0)\cdot M_{\mathrm{W}}(t)\rangle}{3Vk_{\mathrm{B}}T\epsilon_0}. \tag{S8}$$

The final term $\chi_I(\omega)$ contributing to the total susceptibility stems from the ion current

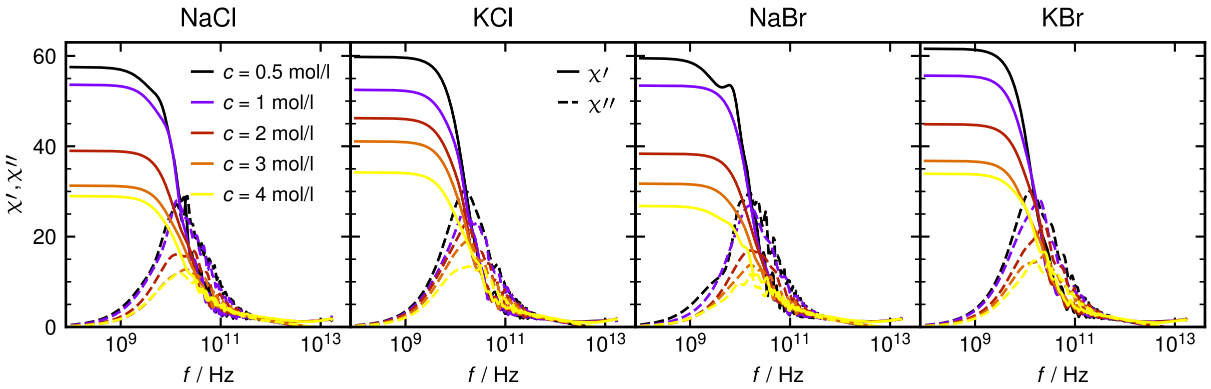

Fig. S7: Dielectric spectra obtained from molecular dynamics simulations of different salt types. Solid lines depict the real parts χ' and dashed lines the imaginary parts χ'', respectively.

auto-correlation function. However, this term diverges at zero frequency due to the finite DC-conductivity of ionic solutions[25]. Thus, we calculate a regularized susceptibility given by,

$$\Delta\chi_{\mathrm{I}}(\omega) = -\frac{i}{\omega}\int_0^\infty (e^{-i\omega t} - 1)\phi_{\mathrm{I}}(t)\,\mathrm{d}t, \tag{S9}$$

with the ion current auto-correlation function defined as

$$\phi_{\mathrm{I}}(t) = \frac{\langle J_{\mathrm{I}}(0)J_{\mathrm{I}}(t)\rangle}{3Vk_{\mathrm{B}}T\epsilon_0}. \tag{S10}$$

Plots of the real (χ') and imaginary (χ'') parts of the total frequency-dependent susceptibility are shown for each ion pair for different concentrations in Fig. S7. As noted in the main text, the dielectric constant used in calculating the dielectric decrement, denoted $\Delta\varepsilon$, is inferred from the lowest frequency point of the real contribution to the total susceptibility for each salt type at every concentration.

S7 Observables for different water models

The mass density, ionic conductivity, and dielectric decrement for water models other than SPC/E are shown in Fig. S8. The values of the water diffusion constant and the dielectric constant at zero ion concentration of the different water models are listed in Table S5.

S8 Comparison to other force fields

The optimal chloride parameters obtained from our optimization are close to the Dang chloride used earlier[17,43]. However, the solvation free energy of the Dang NaCl pair is off by several k_BT, so the present optimization provides a significantly better agreement. The

Table S4: Force field parameters of nonpolarizable water models. For the 4-point water models the charge is on site M.

model	σ (nm)	ε (kJ mol^{-1})	q_H (e)	$r_{\mathrm{OH}}(nm)$	θ_{HOH} (°)	r_{OM} (nm)
SPC/E	0.31656	0.65017	0.4238	0.1	109.47	–
TIP3P	0.31506	0.63638	0.417	0.09572	104.52	–
TIP4P	0.31536	0.64852	0.52	0.09572	104.52	0.15
TIP4P/ε	0.31650	0.76849	0.527	0.09572	104.52	0.105

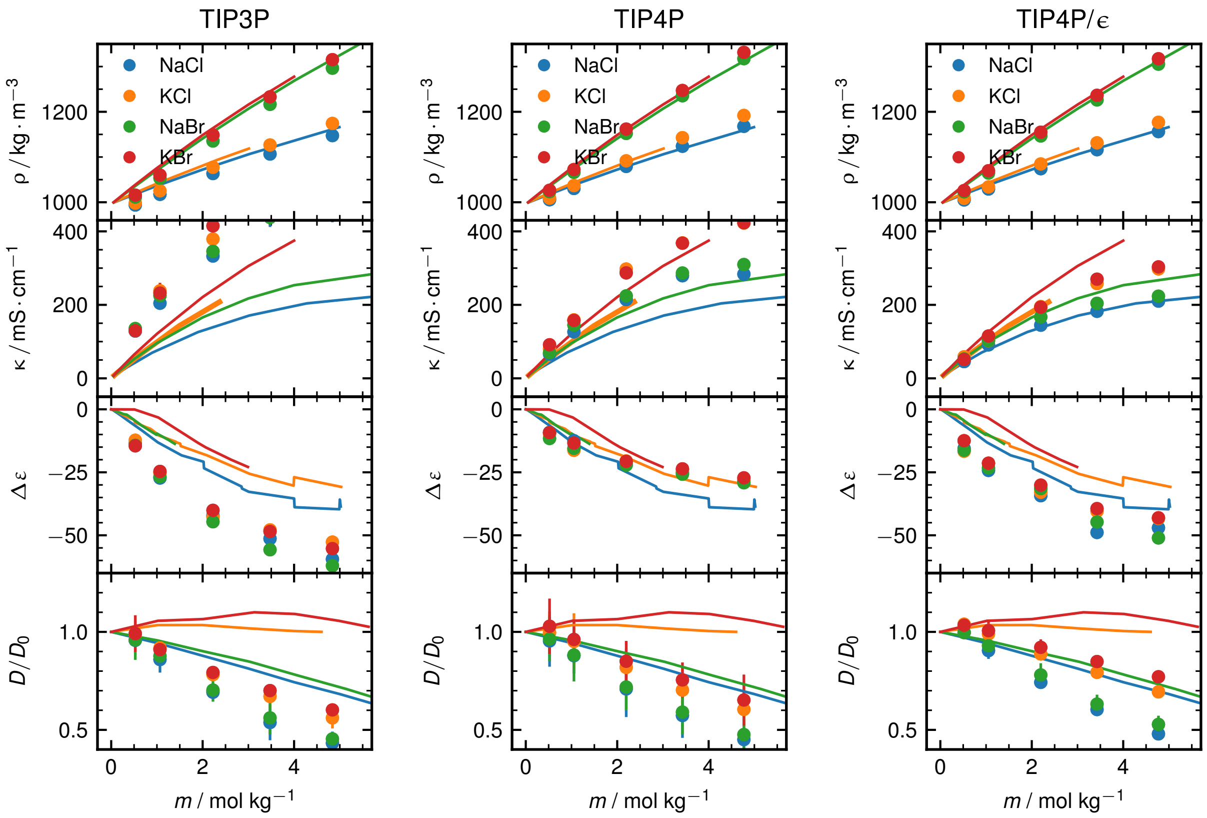

Fig. S8: Observables for the four salts using different water models. From top to bottom: Mass density ρ together with the experimental densities[27,28] (solid lines) as a function of the salt concentration. Ionic conductivities κ together with the experimental conductivities (solid lines)[27,29] as a function of the salt concentration. Dielectric decrement $\Delta\varepsilon = \varepsilon(c) - \varepsilon$ together with the experimental values (solid lines)[30–37]. The zero concentration dielectric constants for the different water models are $\varepsilon_{\mathrm{TIP3P}} = 94$, $\varepsilon_{\mathrm{TIP4P}} = 50$ and $\varepsilon_{\mathrm{TIP4P}/\varepsilon} = 80$[38,39]. Water self-diffusion constant, normalized by the diffusion constant of pure water, together with the experimental data[40]. The pure water diffusion constant D_0 of the different water models is listed in Table S5.

Table S5: The water diffusion constant D and static permittivity ε of nonpolarizable water models. ε is calculated according to Ref. 41. Numbers in brackets denote literature values[38,39,42].

model	D ($\mathrm{nm}^2 \cdot \mathrm{ns}^{-1}$)	ε
SPC/E	2.77 ± 0.05 (2.49)	72.0 (68)
TIP3P	6.01 ± 0.01 (5.19)	92.3 (94)
TIP4P	3.77 ± 0.09 (3.31)	53.9 (50)
TIP4P/ε	2.23 ± 0.01 (2.1)	79.7 (80)

solvation free energy of the CHARMM and Mamatkulov and Schwierz[19] ion pairs is closer to the experimental value, but still off by several k_BT. Because the force field by Mamatkulov and Schwierz[19] is optimized at 300 K with respect to the solvation free energy from Marcus[10] at 298.15 K, a large part of this deviation is caused by the slight differences between the experimental reference values and the temperature difference. This deviation is probably not the primary problem, however. Instead, the strategy employed by Mamatkulov and Schwierz[19] is to pick parameters for the chloride ion in TIP3P which reproduce the single-ion solvation free energy of the Dang chloride in SPC/E water. In focussing on single-ion solvation free energies, their optimization strategy is fundamentally different from our approach. In particular, in contrast to the solvation free energy of ion pairs, single-ion solvation free energies do sensitively depend on the water force field. Relatedly, interface potential differences, which are important for single-ion solvation free energies, also depend on the water force field. Overall, these issues lead to a poor choice of the chloride parameters and the spurious conclusion that ion force fields are not transferable between SPC/E and TIP3P.

References

(1) Latimer, W. M. Single Ion Free Energies and Entropies of Aqueous Ions. *J. Chem. Phys.* **1955**, *23*, 90.

(2) Noyes, R. M. Thermodynamics of Ion Hydration as a Measure of Effective Dielectric Properties of Water. *J. Am. Chem. Soc.* **1962**, *84*, 513–522.

(3) Rosseinsky, D. R. Electrode Potentials and Hydration Energies. Theories and Correlations. *Chem. Rev.* **1965**, *65*, 467–490.

(4) Salomon, M. Thermodynamics of ion solvation in water and propylene carbonate. *J. Phys. Chem.* **1970**, *74*, 2519–2524.

(5) Friedman, H. L.; Krishnan, C. V. In *Aqueous Solutions of Simple Electrolytes*; Franks, F., Ed.; Springer US: Boston, MA, 1973; pp 1–118.

(6) Gomer, R.; Tryson, G. An experimental determination of absolute halfcell emf's and single ion free energies of solvation. *J. Chem. Phys.* **1977**, *66*, 4413.

(7) Wagman, D. D.; Evans, W. H.; Parker, V. B.; Schumm, R. H.; Halow, I.; Bailey, S. M.; Churney, K. L.; Nutall, R. L. The NBS tables of chemical thermodynamic properties: selected values for inorganic and C1 and C2 organic substances in SI units. *J. Phys. Chem. Ref. Data* **1982**, *11*, Supplement No. 2.

(8) Marcus, Y.; Loewenschuss, A. Chapter 4. Standard entropies of hydration of ions. *Annu. Rep. Prog. Chem., Sect. C: Phys. Chem.* **1984**, *81*, 81–135.

(9) Conway, B. E. The Evaluation and Use of Properties of Individual Ions in Solution. *J. Solution Chem.* **1978**, *7*, 721–770.

(10) Marcus, Y. *Ion Properties*; Taylor & Francis, 1997.

(11) Tissandier, M. D.; Cowen, K. A.; Feng, W. Y.; Gundlach, E.; Cohen, M. H.; Earhart, A. D.; Coe, J. V.; R. Tuttle, Jr., T. The Proton's Absolute Aqueous Enthalpy and Gibbs Free Energy of Solvation from Cluster-Ion Solvation Data. *J. Phys. Chem. A* **1998**, *102*, 7787–7794.

(12) Fawcett, W. R. Thermodynamic Parameters for the Solvation of Monatomic Ions in Water. *J. Phys. Chem. B* **1999**, *103*, 11181–11185.

(13) Schmid, R.; Miaha, A. M.; Sapunov, V. N. A new table of the thermodynamic quantities of ionic hydration : values and some applications (enthalpy–entropy compensation and Born radii). *Phys. Chem. Chem. Phys.* **2000**, *2*, 97–102.

(14) Kelly, C. P.; Cramer, C. J.; Truhlar, D. G. Aqueous Solvation Free Energies of Ions and Ion-Water Clusters Based on an Accurate Value for the Absolute Aqueous Solvation Free Energy of the Proton. *J. Phys. Chem. B* **2006**, *110*, 16066–16081.

(15) Marcus, Y. A simple empirical model describing the thermodynamics of hydration of ions of widely varying charges, sizes, and shapes. *Biophys. Chem.* **1994**, *51*, 111–127.

(16) Horinek, D.; Mamatkulov, S. I.; Netz, R. R. Rational design of ion force fields based on thermodynamic solvation properties. *J. Chem. Phys.* **2009**, *130*, 124507.

(17) Bonthuis, D. J.; Mamatkulov, S. I.; Netz, R. R. Optimization of classical nonpolarizable force fields for OH- and H3O+. *J. Chem. Phys.* **2016**, *144*, 104503.

(18) Mamatkulov, S. I.; Rinne, K. F.; Buchner, R.; Netz, R. R.; Bonthuis, D. J. Water-separated ion pairs cause the slow dielectric mode of magnesium sulfate solutions. *J. Chem. Phys.* **2018**, *148*, 222812.

(19) Mamatkulov, S.; Schwierz, N. Force fields for monovalent and divalent metal cations in TIP3P water based on thermodynamic and kinetic properties. *J. Chem. Phys.* **2018**, *148*, 074504.

(20) Lynden-Bell, R. M.; Rasaiah, J. C. From hydrophobic to hydrophilic behaviour: A simulation study of solvation entropy and free energy of simple solutes. *J. Chem. Phys.* **1997**, *107*, 1981–1991.

(21) Latimer, W. M.; Pitzer, K. S.; Slansky, C. M. The Free Energy of Hydration of Gaseous Ions, and the Absolute Potential of the Normal Calomel Electrode. *J. Chem. Phys.* **1939**, *7*, 108–111.

(22) Rashin, A. A.; Honig, B. Reevaluation of the Born model of ion hydration. *J. Phys. Chem.* **1985**, *89*, 5588–5593.

(23) Loche, P.; Ayaz, C.; Schlaich, A.; Bonthuis, D. J.; Netz, R. R. Breakdown of Linear Dielectric Theory for the Interaction between Hydrated Ions and Graphene. *J. Phys. Chem. Lett.* **2018**, *9*, 6463–6468.

(24) Born, M. Volumen und Hydratationswärme der Ionen. *Z. Physik* **1920**, *1*, 45–48.

(25) Rinne, K. F.; Gekle, S.; Netz, R. R. Dissecting ion-specific dielectric spectra of sodium-halide solutions into solvation water and ionic contributions. *J. Chem. Phys.* **2014**, *141*, 214502.

(26) Schröder, C.; Steinhauser, O. *Computational Dielectric Spectroscopy of Charged, Dipolar Systems*; 2010; Publication Title: Computational Spectroscopy: Methods, Experiments and Applications.

(27) Isono, T. Density, viscosity, and electrolytic conductivity of concentrated aqueous electrolyte solutions at several temperatures. Alkaline-earth chlorides, lanthanum chloride, sodium chloride, sodium nitrate, sodium bromide, potassium nitrate, potassium bromide, and cadmium nitrate. *J. Chem. Eng. Data* **1984**, *29*, 45–52.

(28) Gates, J. A.; Wood, R. H. Densities of aqueous solutions of sodium chloride, magnesium chloride, potassium chloride, sodium bromide, lithium chloride, and calcium chloride from 0.05 to 5.0 mol kg-1 and 0.1013 to 40 MPa at 298.15 K. *J. Chem. Eng. Data* **1985**, *30*, 44–49.

(29) Weast, R. C. *CRC Handbook of Chemistry, and Physics, 70th Edition*; CRC Press, 1989.

(30) Hasted, J. B.; Ritson, D. M.; Collie, C. H. Dielectric Properties of Aqueous Ionic Solutions. Parts I and II. *J. Chem. Phys.* **1948**, *16*, 1–21.

(31) Haggis, G. H.; Hasted, J. B.; Buchanan, T. J. The Dielectric Properties of Water in Solutions. *J. Chem. Phys.* **1952**, *20*, 1452–1465.

(32) Barthel, J. *Electrolyte Data Collection: Dielectric Properties of Water and Aqueous Electrolyte Solutions (Chemistry Data Series)*; Dechema, 1996.

(33) Chen, T.; Hefter, G.; Buchner, R. Dielectric Spectroscopy of Aqueous Solutions of KCl and CsCl. *J. Phys. Chem. A* **2003**, *107*, 4025–4031.

(34) Wachter, W.; Kunz, W.; Buchner, R.; Hefter, G. Is There an Anionic Hofmeister Effect on Water Dynamics? Dielectric Spectroscopy of Aqueous Solutions of NaBr, NaI, NaNO3, NaClO4, and NaSCN. *J. Phys. Chem. A* **2005**, *109*, 8675–8683.

(35) Abascal, J. L. F.; Vega, C. A general purpose model for the condensed phases of water: TIP4P/2005. *J. Chem. Phys.* **2005**, *123*, 234505.

(36) Levy, A.; Andelman, D.; Orland, H. Dielectric Constant of Ionic Solutions: A Field-Theory Approach. *Phys. Rev. Lett.* **2012**, *108*, 227801.

(37) Shcherbakov, V. V.; Artemkina, Y. M.; Korotkova, E. N. Dielectric properties and high-frequency conductivity of the sodium chloride-water system. *Russ. J. Inorg. Chem.* **2014**, *59*, 922–926.

(38) Vega, C.; Abascal, J. L. F. Simulating water with rigid non-polarizable models: a general perspective. *Phys. Chem. Chem. Phys.* **2011**, *13*, 19663–19688.

(39) Fuentes-Azcatl, R.; Alejandre, J. Non-Polarizable Force Field of Water Based on the Dielectric Constant: TIP4P/epsilon. *J. Phys. Chem. B* **2014**, *118*, 1263–1272.

(40) Müller, K. J.; Hertz, H. G. A Parameter as an Indicator for Water-Water Association in Solutions of Strong Electrolytes. *J. Phys. Chem.* **1996**, *100*, 1256–1265.

(41) Neumann, M. Dipole moment fluctuation formulas in computer simulations of polar systems. *Molecular Physics* **1983**, *50*, 841–858.

(42) Mahoney, M. W.; Jorgensen, W. L. Diffusion constant of the TIP5P model of liquid water. *J. Chem. Phys.* **2000**, *114*, 363–366.

(43) Fyta, M.; Netz, R. R. Ionic force field optimization based on single-ion and ion-pair solvation properties: Going beyond standard mixing rules. *J. Chem. Phys.* **2012**, *136*, 124103.
